# Classification for Longevity Potential: The Use of Novel Biomarkers

**DOI:** 10.3389/fpubh.2016.00233

**Published:** 2016-10-28

**Authors:** Marian Beekman, Hae-Won Uh, Diana van Heemst, Manfred Wuhrer, L. Renee Ruhaak, Vanessa Gonzalez-Covarrubias, Thomas Hankemeier, Jeanine J. Houwing-Duistermaat, P. Eline Slagboom

**Affiliations:** ^1^Molecular Epidemiology, Leiden University Medical Center, Leiden, Netherlands; ^2^Medical Statistics and Bioinformatics, Leiden University Medical Center, Leiden, Netherlands; ^3^Gerontology and Geriatrics, Leiden University Medical Center, Leiden, Netherlands; ^4^Center for Proteomics and Metabolomics, Leiden University Medical Center, Leiden, Netherlands; ^5^Department of Translational Molecular Pathology, MD Anderson Cancer Center, Houston, TX, USA; ^6^Analytical Biosciences, Leiden Academic Centre for Drug Research, Leiden, Netherlands; ^7^Instituto Nacional de Medicina Genomica (INMEGEN), Mexico City, Mexico; ^8^Netherlands Metabolomics Centre, Leiden, Netherlands

**Keywords:** human longevity potential, classification and prediction, Framingham Risk Score, biomarker, sex-specific analysis

## Abstract

**Background:**

In older people, chronological age may not be the best predictor of residual lifespan and mortality, because with age the heterogeneity in health is increasing. Biomarkers for biological age and residual lifespan are being developed to predict disease and mortality better at an individual level than chronological age. In the current paper, we aim to classify a group of older people into those with longevity potential or controls.

**Methods:**

In the Leiden Longevity Study participated 1671 offspring of nonagenarian siblings, as the group with longevity potential, and 744 similarly aged controls. Using known risk factors for cardiovascular disease, previously reported markers for human longevity and other physiological measures as predictors, classification models for longevity potential were constructed with multiple logistic regression of the offspring-control status.

**Results:**

The Framingham Risk Score (FRS) is predictive for longevity potential [area under the receiver operating characteristic curve (AUC) = 64.7]. Physiological parameters involved in immune responses and glucose, lipid and energy metabolism further improve the prediction performance for longevity potential (AUCmale = 71.4, AUCfemale = 68.7).

**Conclusion:**

Using the FRS, the classification of older people in groups with longevity potential and controls is moderate, but can be improved to a reasonably good classification in combination with markers of immune response, glucose, lipid, and energy metabolism. We show that individual classification of older people for longevity potential may be feasible using biomarkers from a wide variety of different biological processes.

## Introduction

The aging process is underlying the physiological and functional decline of the body with time. In older people, the heterogeneity of bodily decline and the ability to cope with exposure and resiliency is increasing and chronological age may then not be the best predictor of disease risk and mortality. Biomarker studies for the bodily functional decline aim to develop a measure for biological age as a better marker for the residual lifespan and mortality.

In large population-based studies, several measures of physiological dysregulation (1–3) and biological age scores (4–6) have been developed using cross sectional and longitudinal data. The challenge in biomarker development lays in the prediction of residual lifespan or mortality risk for an individual. The Framingham Risk Score (FRS) (7) that provides an estimate of the individual 10-year cardiovascular disease risk, and is used in the prediction of cardiovascular health as a tool to assess optimal cardiovascular treatment, is the most successful biomarker for cardiovascular disease risk.

If prediction of residual lifespan at an individual’s level would be possible, it should be possible to classify people as those with an expected longer residual lifespan or those with an expected shorter residual lifespan. A group of people prone to become long-lived are members of long-lived families. They seem to escape, delay the onset, or exhibit reduced severity of age-related disease. They also have ~30% survival advantage as compared to their birth cohort (8, 9). Offspring of long-lived parents show lower prevalence of type 2 diabetes, hypertension, and myocardial infarction than control groups as well as a delayed onset of cardiovascular disease and they have, despite similar cancer prevalence, a lower cancer-specific mortality (10, 11). This indicates that at similar age offspring of long-lived parents are healthier than the general population (12, 13) and that mechanisms involved in the escape, delay of onset of disease and/or the severity of disease may potentially be discovered by studying familial longevity. The spouses of such members of long-lived families may represent the general population (controls) and have an average expected residual lifespan. The ability to classify people as member of a long-lived family or as control could provide insight in the feasibility of a biomarker for residual life or mortality.

Here, we aim to predict whether a person belongs to a long-lived family, as a proxy for longevity potential, or not. Therefore, as part of the Leiden Longevity Study (8, 13), we extensively phenotyped 1671 offspring of nonagenarian siblings and 744 of their partners as controls from the general population and we performed statistical modeling. First, we determined to what extent the established FRS predicts longevity potential. Next, we examined whether this prediction by the FRS could be improved by the markers that are known to be different between controls and members of long-lived families: serum levels of glucose (14), insulin (14), free triiodothyronine (15), triglycerides (14), adiponectin (16), and the ratio of total cholesterol over high-density lipoprotein cholesterol (HDL-C) (17), and low-density lipoprotein (LDL) particle size (18, 19). At last, the effect of other phenotypic measures, such as cell counts and IgG glycosylation, on the prediction for longevity potential was explored.

## Materials and Methods

### The Leiden Longevity Study

Between 2002 and 2006, the Leiden Longevity study included 421 Caucasian families consisting of long-lived siblings together with their offspring and the partners of the offspring (8). Long-lived families were recruited if at least two long-lived siblings were alive and willing to participate. Men were considered long-lived if 89 years or older and females 91 years or older. In 2001, less than 0.5% of the Dutch population fulfilled these criteria. In total, 2415 members of long-lived families have been recruited consisting of the offspring of long-lived siblings (*n* = 1671) and the partners of the offspring as controls (*n* = 744). The Medical Ethical Committee of the Leiden University Medical Centre approved the study and informed consent was obtained from all subjects. All procedures performed in studies involving human participants were in accordance with the ethical standards of the institutional and/or national research committee and with the 1964 Helsinki declaration and its later amendments or comparable ethical standards.

Non-fasted venous blood samples were taken at baseline. Between November 2006 and May 2008, additional information on self-reported smoking habits, weight, and height was collected for the offspring and the controls, and information on medical history for hypertension, type 2 diabetes, and cardiovascular disease was re-quested from the participants’ treating physicians (13).

### Serum and Plasma Parameters

#### Grouping of Measures

We considered the components of the FRS as the “*cardiovascular risk factors*” (7), which are current smoking habits, prevalence of hypertension, type 2 diabetes, plasma levels of total cholesterol, HDL-C, and low-density lipoprotein cholesterol (LDL-C). As “*Longevity markers*” we considered molecular measures that have been reported to be different between offspring of long-lived individuals and controls, which are plasma levels of glucose (14), insulin (14), free triiodothyronine (fT3) (15), triglycerides (14), adiponectin (16), the ratio of total cholesterol over HDL-C (Total/HDL ratio) (17), and LDL particle size (18, 19). Because a lot of other parameters have been determined in participants of the LLS, we also define a group of “*other molecular markers*” that are white blood cell count, hemoglobin, hematocrit, platelet count, neutrophil count, lymphocyte count, monocyte count, eosinophil count, basophil count, count of large unstained cells, IgG glycosylation species, plasma levels of IGF1, IGF1BP3, fructosamine, free thyroxine (fT4), thyroid-stimulating hormone (TSH), high-sensitive C reactive protein (HsCRP), homocystein, Il6, free fatty acids (FFA), 25(OH) vitamin D3, ApoE, and leptine. Furthermore, height, weight, BMI, HDL particle size (HZ), *APOE* isoform, rs405509 (APOE −219 A/C), and cytomegalovirus (CMV) serostatus have been determined. All serum measurements were performed using fully automated equipment and all chemical analyses were performed in a single batch at the Department of Clinical Chemistry, Leiden University Medical Center, the Netherlands. Table S1 in Supplementary Material shows the descriptives of all analyzed parameters.

##### Cardiovascular Risk Factors

Current smoking habits were self-reported through a questionnaire to the study participants, while prevalence of hypertension and type 2 diabetes has been reported by their general practitioner. For total cholesterol, HDL-C, and triglyceride levels the Hitachi Modular P 800 from Roche (Almere, the Netherlands) was used. CVs of these measurements were less than 5%. For LDL-C, the Friedewald formula was used.

##### Known Longevity Markers

For serum glucose levels the Hitachi Modular P 800 from Roche (Almere, the Netherlands) was used. CVs of these measurements were less than 5%. For insulin levels the Immulite 2500 from DPC (Los Angeles, CA, USA) was used. The coefficient of variation (CV) for this measurement was less than 8%. For free triiodothyronine the Modular E170 was used (Roche, Almere, the Netherlands). The coefficients of variation of these measurements were all below 5% (15). Adiponectin (R&D Systems Europe, Ltd., Abingdon, UK) was determined using specific sandwich enzyme-linked immunosorbent assay (ELISA) and triglyceride levels and LDL particle sizes and concentrations were measured using proton nuclear magnetic resonance (NMR) spectroscopy (LipoScience Inc., Raleigh, NY, USA) (20).

##### Other Molecular Markers

For insulin-like growth factor-1 (IGF-1) and insulin-like growth factor binding protein 3 (IGFBP3) levels the Immulite 2500 from DPC (Los Angeles, CA, USA) was used. The CV for this measurement was less than 8%. For thyrotropin and free thyroxine the Modular E170 was used, for hsCRP the Cobas Integra 800 was used (Roche, Almere, the Netherlands). The CVs of these measurements were all below 5% (15). Levels of fructosamine (millimolar/liter) have been determined on a Roche Integra analyzer, using nitroblue tetrazolium reagent (Roche Diagnostics, Mannheim, Germany), and FFA were measured using NEFA–HR2 kits (Wako Chemicals GmbH, Neuss, Germany) on the Roche/Hitachi Modular P800 analyzer (Roche Diagnostics) The CVs for this measurement were below 4%. Homocystein levels were determined using a competitive immunoassay (Architect) (21). Plasma levels of Apoliporotein E, interleukin 6 (IL6) and leptin were determined using specific sandwich ELISA (22). Levels of 25(OH) vitamin D3 were determined on the Cobas e 411 analyzer (Roche, Almere, the Netherlands).

Furthermore, automated white blood cell differential (counts of neutrophils, lymphocytes, monocytes, eosinophils, basophils, and large unstained cells) were determined as well as hemoglobin, hematocrit, and platelet count at the Department of Clinical Chemistry, Leiden University Medical Center, the Netherlands.

For the current analyses, we used self-reported measures of height and weight by which body mass index was determined [(weight in kg)/(height in cm)^2^]. The CMV serostatus was determined by the CMV IgG kit (ETI-CYTOK-G PLUS DiaSorin, Saluggia, Italy) based on enzyme immunoassay technology.

To determine the *APOE* isoform two SNPs have been genotyped, in addition to rs405509, which located in the *APOE* promotor and has previously been associated the development of dementia, using custom made Taqman Assyas (Applied Biosystems).

High-density lipoprotein particle sizes were measured using proton NMR spectroscopy (LipoScience Inc, Raleigh, NY, USA) (19).

Glycosylation of IgG was measured in citrate plasma with mass spectrometry as described in Ruhaak et al. (23). In short, immunoglobulin G was purified from citrate plasma samples in 22 96-well filter plates using a Protein A affinity purification step. Two microliters of plasma were added to 15 μl Protein A coated beads in 185 μl PBS in a 96-well plate, and incubated at room temperature for 1 h. After washing, IgGs were eluted using 100 mM formic acid. After tryptic digestion of the isolated IgGs, glycopeptides were purified using a C_18_-SPE plate. Consequently, large-scale analysis of IgG glycosylation profiles was performed using MALDI-TOF-MS. Nomenclature of glycoforms is as follows: IgG1 G0 (IgG1A), IgG1 G1 (IgG1 B), IgG1 G2 (IgG1 C), IgG1 G0N (IgG1 D), IgG1 G1N (IgG1 E), and IgG1 G2N (IgG1 F).

Lipidome analysis was performed in citrate plasma by ultra-high pressure liquid chromatography coupled to mass spectrometry (UPLC-MS) using an optimized version of the method reported by Hu et al. (24) and has been described previously (25). Validation parameters were: linearity LPC (19:0), *r*^2^ > 0.99; PC (34:0), *r*^2^ > 0.97; PE (34:0), *r*^2^ > 0.98; TG (45:0), *r*^2^ > 0.99; repeatability and reproducibility, RSD < 15%. Two freeze–thaw cycles did not alter validation parameters (RSD < 15%). Lipid names and abbreviations were assigned according to Lipid Maps nomenclature (http://www.lipidmaps.org).

### Statistical Methods

Since missing values occur in various variables and individuals (Table S1 in Supplementary Material), we used the multivariate imputation by chained equations (MICE) (26) method to impute the missing values, and to fully use available data. We created five imputed complete data sets, analyzed each data set separately, and combined the results as described in Chapter 3 of Multiple imputation for non-response in surveys by Rubin (27).

To assess association between longevity potential (offspring-controls allocation) and the phenotypic parameters, logistic regression models were fitted. Since the aging process seems different between the two sexes (28), we performed the current analyses stratified by sex and adjusted for age. The association of the FRS, of which age is a component, with offspring-control allocation can be interpreted that it is at least not due to the age difference between the two groups. Furthermore, within-family (between-siblings) dependence is taken into account by using generalized estimating equations (GEE) approach with robust SE estimates of the effect sizes.

Prediction models for longevity potential are constructed with multiple logistic regression of the offspring-partners status on a number of published parameters as predictors: the (known) cardiovascular risk factors and longevity markers, FRS. Since many other molecular phenotypes were available, we investigated which set of predictors jointly predict the longevity outcome best. We constructed the various age adjusted models starting with FRS as a surrogate variable for cardiovascular risk factors; FRS as a compulsory variable (Model 1), FRS as a compulsory variable with addition of longevity markers (Model 2), FRS as a compulsory variable with addition of other molecular phenotypes (Model 3). Additionally, instead of using FRS, we constructed the model based on all markers, which include the components of cardiovascular risk factors (Model 4):

Model 1: Group = β_1_ × FRS + β_2_ × Age.
Model 2: Group = β_1_ × FRS + β_2_ × Age + β_3–9_ × Known Longevity Markers.
Model 3: Group = β_1_ × FRS + β_2_ × Age + β_3–9_ × Known Longevity Markers + β_10–59_ Other Molecular Markers.
Model 4: Group = β_1_ × Age + β_2–8_ × Cardiovascular Risk Factors + β_9–15_ Known Longevity Markers + β_16–65_ × Other Molecular Markers.

The selection algorithm based on elastic net (29) was implemented, in order to deal with possible correlations between the parameters and to select relevant (or important) variables. This method is a combination of lasso (for selection of predictors) and ridge (for shrinkage) logistic regression, and encourages grouping effect. The original data sets were divided into two sets: (1) for building prediction model and (2) for validation of the selected model. Available families and controls were both randomly divided into two equally sized halves. Half of the first set consisted of randomly selected families and half of randomly selected controls, so that by 10-fold cross validation the best predicting model was constructed. The second set contained the other half of families and the other half of controls, and was used for validation of the selected model. Finally, prediction performance of these various models was compared using area under the receiver operating characteristic (ROC) curve (AUC) based on validation sets. To assess relative importance of the individual markers in the selected model, Breiman’s Random Forests (30) was used.

Data analyses are performed using the freely available packages and software R version 2.13.1 (R Development Core Team, 2009).

## Results

### Study Population

Table 1 provides the descriptives of the studied groups with longevity potential and controls from the Leiden Longevity Study. While the males with longevity potential (mean age 59.3 years) were on average younger than male controls (mean age 61.2 years), the females with longevity potential (mean age 59.4 years) were older than female controls (mean age 56.9 years). Because of the presence of this mean age difference between the groups with longevity potential and controls, all subsequent analyses were adjusted for age.

**Table 1:** **Characteristics of study population (mean and SD between brackets) stratified by sex and offspring-control allocation**.

Characteristics	Reference^b^	Male		Female	
		Offspring	Controls	Offspring	Controls
Number of individuals		772	315	899	429
Age (years)		59.3 (6.58)	61.2 (7.46)	59.4 (6.47)	56.9 (6.95)
BMI index		25.7 (2.96)	25.8 (3.23)	25.0 (4.01)	25.4 (3.88)
**Cardiovascular risk factors**					
Smoking YES *N* (%)	(7, 31)	88 (13.8)	42 (15.0)	98 (12.8)	58 (15.8)
Hypertension YES *N* (%)	(7, 31)	148 (22.8)	73 (27.1)	171 (22.3)	105 (28.5)
Diabetes YES *N* (%)	(7, 31)	32 (4.8)	27 (10.1)	28 (3.6)	20 (5.4)
Total cholesterol (mmol/l)	(31)	5.46 (1.12)	5.47 (1.12)	5.68 (1.24)	5.73 (1.15)
HDL-C (mmol/l)	(7)	1.27 (0.37)	1.23 (0.35)	1.60 (0.45)	1.56 (0.49)
LDL-C (mmol/l)	(7)	3.30 (0.94)	3.30 (0.95)	3.38 (1.02)	3.39 (0.94)
Framingham risk score	(7)	10.96 (6.61)	13.12 (7.93)	4.17 (2.33)	4.47 (3.00)
**Longevity markers**					
Glucose (mmol/l)	(14)	5.91 (1.38)	6.40 (2.20)	5.72 (1.22)	5.90 (1.41)
Insulin^a^ (mmol/l)	(14)	17.48 (2.26)	19.32 (2.36)	14.75 (2.23)	16.61 (2.25)
Free triiodothyronine (pmol/L)	(15)	4.31 (0.66)	4.30 (0.67)	3.89 (0.69)	4.02 (0.85)
Triglyceride^a^ (mmol/l)	(14)	1.74 (1.71)	1.85 (1.72)	1.36 (1.66)	1.50 (1.71)
Total/HDL ratio	(17)	4.57 (1.42)	4.78 (1.58)	3.77 (1.21)	3.98 (1.38)
LDL particle size (nm)	(18, 19)	20.97 (0.78)	20.82 (0.76)	21.58 (0.75)	21.45 (0.80)
Adiponectine (mg/l)	(16)	4.92 (2.25)	4.66 (2.15)	7.65 (3.44)	7.13 (3.65)

*^a^Natural logarithmically back transformed means*.

*^b^References refer to published literature demonstrating that the measure is indeed a biomarker for cardiovascular risk or longevity potential*.

*BMI index, body mass index; HDL-C, high-density lipoprotein cholesterol; LDL-C, low-density lipoprotein cholesterol; Total/HDL-C, the ratio of total cholesterol level over HDL-cholesterol level; LDL size, low-density lipoprotein particle size; SE, standard error of the estimate of the effect size log-OR*.

With regard to the cardiovascular risk factors the prevalence of type 2 diabetes was lower among males with longevity potential (*P*-value = 0.007) as compared to male controls. In the comparison of females with longevity potential and controls, those with longevity potential exhibited lower prevalence of hypertension (*P*-value = 0.001) (Table 2).

**Table 2:** **Univariate association of cardiovascular risk factors and longevity markers with longevity as offspring-control allocation in the Leiden Longevity Study**.

	Male *N* = 1087			Female *N* = 1328		
	log-OR^a^	SE	*P*-value	log-OR^a^	SE	*P*-value
Age	−0.04	0.01	**3.71 × 10^−4^**	**0.06**	**0.01**	**1.00 × 10^−8^**
**Cardiovascular risk factors**						
Smoking	−0.21	0.21	0.316	−0.12	0.19	0.536
Hypertension	−0.14	0.17	0.422	−0.49	0.15	**0.001**
Diabetes	−0.75	0.28	**0.007**	−0.56	0.31	0.069
Total cholesterol	−0.02	0.06	0.749	−0.06	0.05	0.230
HDL-C	0.39	0.20	0.050	0.23	0.14	0.119
LDL-C	−0.03	0.08	0.697	−0.04	0.07	0.585
**Longevity markers**						
Glucose	−0.16	0.04	**1.28 × 10^−4^**	−0.14	0.05	**0.003**
Insulin	−0.13	0.09	0.140	−0.23	0.08	**0.004**
Free triiodothyronine	−0.06	0.11	0.564	−0.21	0.09	0.023
Triglyceride	−0.09	0.05	0.059	−0.28	0.07	**3.18 × 10^−5^**
Total/HDL-C	−0.11	0.05	0.025	−0.16	0.05	**0.002**
LDL size	0.23	0.09	0.013	0.26	0.08	**0.002**
Adiponectin	0.07	0.03	0.035	0.04	0.02	**0.039**

*^a^Effects of the risk factors (except age) are adjusted for age. A log-OR below 0 indicates that offspring as compared to controls show lower prevalence (smoking, hypertension, diabetes) or serum levels of the associated trait. A log-OR above 0 indicates that offspring as compared to controls show higher serum levels of the associated trait*.

*HDL-C, high-density lipoprotein cholesterol; LDL-C, low-density lipoprotein cholesterol; Total/HDL-C, the ratio of total cholesterol level over HDL-cholesterol level; LDL size, low-density lipoprotein particle size, SE, standard error of the estimate of the effect size log-OR*.

*Values in bold are significant *P*-values adjusted for multiple testing [Bonferroni significant *P*-value < (0.05/7) = 0.0071]*.

There are biomarkers that are described to discriminate between members of long-lived families, as individuals with longevity potential, and similarly aged controls, which we will further call “known longevity markers” (18, 19). Table 2 shows the sex-specific analyses for these known longevity markers illustrating that associations seem not sex-specific and that directions of association are similar in both sexes. Glucose levels were lower among males with longevity potential as compared to male controls (*P*-value = 1.28 × 10^−4^). In the comparison of females with longevity potential and female controls glucose levels (*P*-value = 0.003), insulin levels (*P*-value = 0.004), triglyceride levels (*P*-value = 3.18 × 10^−5^), and ratios of total cholesterol over HDL cholesterol (*P*-value = 0.002) were lower in females with longevity potential, while LDL particle size (*P*-value = 0.002) was higher in females with longevity potential (Table 2).

### Prediction of Longevity Potential by FRS

In order to classify the Leiden Longevity Study participants into those with longevity potential and controls based on the FRS, we first imputed the missing values using MICE (26) methodology so that the full data set is available for analyses. Using the five imputed data sets (see Materials and Methods for details), we calculated the FRS (7) from the separate cardiovascular risk factors. Since FRS can only be calculated on people that have not yet had a cardiovascular attack, we were able to calculate FRS in 2301 individuals (out of 2415), i.e., 716 males with longevity potential (out of 772), 300 male controls (out of 315), 873 females with longevity potential (out of 899), and 412 female controls (out of 429).

Males with longevity potential have lower FRS (FRS_mean_ = 10.9) than male controls (FRS_mean_ = 13.1) (*P*-value = 0.008). Females with longevity potential also have lower cardiovascular risk scores (FRS_mean_ = 4.2) than female controls (FRS_mean_ = 4.5; *P*-value = 0.003). The significance of the mean difference is adjusted for age difference.

The dataset, including 2301 individuals with complete data regarding the FRS, is next being used for the prediction of longevity potential. First, a multiple logistic regression model, including the cardiovascular risk factors and known longevity markers as predictors was fitted in order to evaluate the partial contribution of each risk factor in the presence of the others. It appeared that only glucose levels in males (*P*-value = 0.004) and the prevalence of hypertension in females (*P*-value = 0.006) have significant partial contribution to the joint model (Table S2 in Supplementary Material).

Next, we investigated whether FRS is able to predict longevity potential, and whether the addition of known longevity markers will improve the prediction. In order to evaluate the performance of different prediction models, the ROC and the area under the ROC curve (AUC) were computed. To validate the predictive value of this prediction model, the ROC and the AUC were computed using the validation data sets (Table 3). The AUCs for the male and female prediction models based on the FRS only (Model 1) were 64.7 and 64.7, respectively. Given the fact that our design is case-cohort and there are a limited number of predictors, the moderate estimate of the area under the curve is expected and, therefore, our results indicate that the FRS is to some extent predictive for longevity potential. If the model was extended with known longevity markers (Model 2), the AUC further increased to 65.6 for males and 66.4 for females (Table 3).

**Table 3:** **Predictive value (AUC) based on validation sets of various prediction models**.

Prediction model	Male	Female
Model 1^a^: FRS	64.7	64.7
Model 2^a^: FRS + known longevity markers	65.6	66.4
Model 3^a^: FRS + known longevity markers + other molecular markers	71.4	68.7
Model 4^b^: All markers	70.8	68.5

*^a^Age and Framingham risk score (FRS) were forced to be selected in the model*.

*^b^Age only was forced to be selected in the model*.

**Figure 1:**
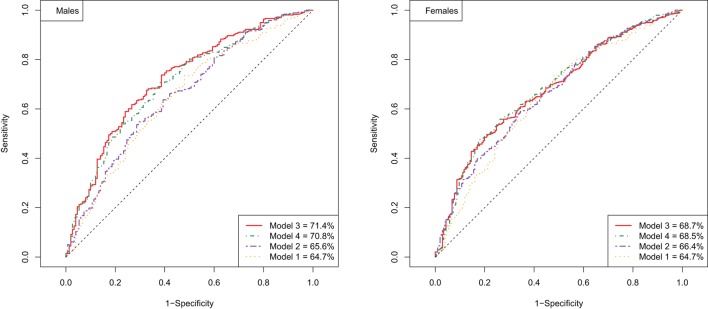
**ROC curve of prediction models for longevity potential in males (left panel) and females (right panel)**.

Next, we explored if there are additional parameters from our data that can further improve the predictive model for longevity potential. Specifically, we test whether these additional parameters could contribute to (or improve) the best prediction model based on the known cardiovascular risk factors and known longevity markers. We extended the prediction models with additional predictors that are selected based on the cross-validated elastic net. Each of five imputed data sets was divided into two sets of equal size: for building the prediction model and for validation of the selected model. The addition of 63 traits (Table S1 in Supplementary Material) (Model 3), among others white blood cell counts, IgG glycosylation, and lipidomic traits, measured in blood did further increase the AUC in male to 71.4 and in women to 68.5 (Figure 1). The prediction of the longevity potential was not further improved when the separate cardiovascular risk factors, instead of the FRS, were tested in addition to the known longevity markers and all other molecular markers (Model 4) (Table 3).

**Figure 2:**
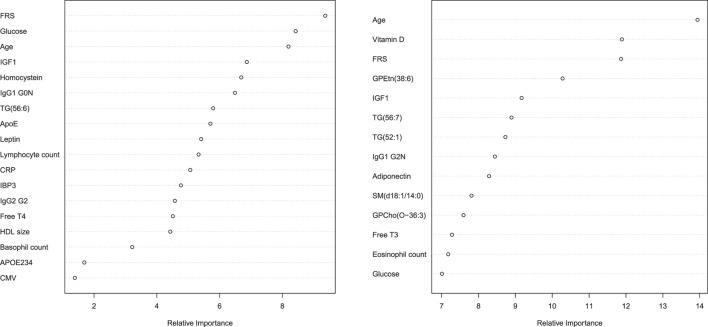
**Relative importance plot in the prediction longevity potential in males (left panel) and females (right panel) in model 3**. The *x*-axis shows the relative importance, as mean decrease accuracy, and the *y*-axis shows the traits that have been selected in model 3. The importance plot is a critical output of the random forest algorithm and to assess relative importance of the individual markers in the selected model, the machine learning tool Breiman’s Random Forests^21^ was used. The traits on the *y*-axis ordered top-to-bottom as most- to least-important. Therefore, the most important variables for classification are at the top and an estimate of their importance is given by the position of the dot on the *x*-axis. Abbreviations left panel: FRS, Framingham risk score; IGF1, insulin-like growth factor 1; IgG1 G0N, immune globulin subclass G1 with Fc *N*-glycosylation at G0; TG (56:6), triglyceride with 56 carbon atoms and 6 double bonds; ApoE, lipoprotein E plasma levels; CRP, C-reactive protein plasma levels; IBP3, IGF1-binding protein 3; IgG2 G2, immune globulin subclass G2 with Fc *N*-glycosylation at G2; Free T4, Thyroxine; HDL size, high-density lipoprotein particle size; APOE234, Apolipoprotein isoform; CMV, cytomegalovirus seropositivity. Abbreviations right panel: FRS, Framingham risk score; GPEtn (38:6), ethanolamine glycerophospholipid with 38 carbon atoms and 6 double bonds; IGF1, insulin-like growth factor 1; TG(56:7), triglyceride with 56 carbon atoms and 7 double bonds; TG(52:1), triglyceride with 52 carbon atoms and 1 double bond; IgG1 G2N, immune globulin subclass G2 with Fc *N*-glycosylation at G2; SM(d18:1/14:0), Sphingomyelin (d18:1/14:0); GPCho(O-36:3), phosphatidylcholine O-36:3; Free T3, triiodothyronine.

The parameters that were selected for the optimal prediction of longevity potential (Model 3) in each of the five imputed data sets, while age and FRS were forced into the model, are shown in Figure 2. We observe that parameters involved in glucose metabolism (e.g., glucose, igf1 levels), lipid metabolism (f.e. triglycerides, sphingomyelins, lipoprotein particle size, *APOE*234 genotype), energy metabolism (f.e. thyroid hormones, leptin, adiponectin), and the immune system (f.e. IgG glycosylation, HsCRP, blood cell counts) play an important role in the prediction of longevity potential in both males and females.

## Discussion

In this paper, we aimed at classification of older people as those with longevity potential or without. People with longevity potential were represented by offspring of nonagenarian siblings and compared to their partners as those with an expected average lifespan. The FRS, as an indicator of cardiovascular health, is a modest classifier for middle aged persons with longevity potential. Moreover, physiological parameters involved in immune response and glucose, lipid, and energy metabolism further improve the prediction value for longevity potential. We concluded that we are reasonably able to classify older people as controls or those with longevity potential.

The magnitude of the predictive value of ~0.70 indicates that 70% of the older people are classified in the right group. We expected that the classifier for being a member of a long-lived family, as proxy for longevity potential, is not able to reach 100% accuracy, since the predictive value of a classifier for a polygenic disease with a low frequency is expected to be very poor (32). In addition, the longevity phenotype is known for its complexity due to its multifactorial architecture. Also, the controls in our study design reflect population controls of who we do not know whether they will become long-lived. Therefore, the groups to classify are heterogeneous, i.e., among those with longevity potential there are people who will not become long-lived and among the controls there are people that will become long-lived. Therefore, we conclude that an AUC of about 70 is fairly high in predicting longevity potential.

It is known that in the comparison offspring of long-lived people and their spouses, there is a clear underestimation of the contrast. A Danish cohort study showed that spouses of descendants of long-lived parents also show lower mortality than age and sex-matched population controls (33). This suggests that, due to the not as optimum contrast in longevity potential in the current study, the classification potential is also underestimated. From this, we may conclude that if a study with the optimum contrast will be investigated, it will probably classify better, which increases the feasibility of developing a predictor for residual life or mortality at an individual level.

A limitation of our study is that the glycosylation measures as well as the lipidomics measures have more that 5% missing values. We used MICE to impute the missing values in five replicate imputed data sets to minimize the influence of the phenotype imputation. Though, we should be carefully interpreting the influence of the specific traits.

Interestingly, the addition of the “other molecular markers” to the prediction model seems to contribute considerably the prediction performance (model 3 vs. model 2), and even seem to increase the performance more than the “known longevity markers.” Potentially there seems to be redundancy in biomarker signal between FRS markers and “known longevity markers,” such as the lipid markers. In the relative importance plot, it becomes clear that in men indeed other than lipid-based markers are selected in the model to predict longevity potential, such as IgG glycosylation measures and *APOE*234 genotype. In females, it seems that very specific lipids, others than the classical measures, extra contribute to the prediction model for longevity potential, such as levels of sphingomyelin (d18:1/14:0) and phosphatidylcholine O-36:3. Notable is that the selected predicting parameters reflect different aspects of aging, implying that if additional biomarkers for different biological processes may even further improve the prediction value. For example, from the nine hallmarks of aging (34), we potentially covered three hallmarks: altered intracellular communication (IgG glycosilation), deregulation of nutrient sensing (glucose and lipid metabolism) and mitochondrial dysfunction (thyroid metabolism). Because physiological dysregulation likely occur at multiple biological processes (35, 36), future identified biomarkers of the other six hallmarks, such as stem cell exhaustion, genomic instability, and epigenetic alterations, have high potential to improve the prediction performance.

Previously, in population-based studies, estimations of physiological dysregulation or biological age have been associated with health outcomes and mortality (2, 3, 5). The difficulty in the comparison of each of these studies is that different study cohorts have been used and that different parameters have been determined to be included in analyses. Previously, albumin has been determined as an important predictor of biological age and residual lifespan (37, 38), which should encourage cohorts to measure metabolomics platforms that include albumin levels. Ultimately, transcriptomics, proteomics, and metabolomics measures should be harmonized for human cohorts and then added to the models for biological age and physiological dysregulation and investigate generalizability over multiple studies.

The next step before use in the clinic would be to test whether resulting established biomarkers of residual lifespan, mortality, and longevity potential are improving after a beneficial lifestyle intervention in older people. To delay the onset of age-related disease, older people are encouraged changing their lifestyle by adjusting their food and increasing their physical activity (39). To monitor whether the lifestyle intervention is not harmful for the older individual or initiate physiological dysregulation, the new biomarkers for longevity potential or biological age could be developed as a monitoring tool.

In conclusion, the classification of older people into groups with longevity potential and controls is moderate using the FRS. To acquire reasonably good classification markers of immune response, glucose, lipid, and energy metabolism are required. To improve the classification of older people according to longevity potential, novel biomarkers are required representing most likely additional biological processes, such as stem cell exhaustion, genomic instability, and epigenetic alterations. We show that individual classification of older people for longevity potential may be feasible using biomarkers from a wide variety of different biological processes.

## Author Contributions

MB, H-WU, JH-D, and PS designed the work in this paper and contributed to the interpretation of the results; MB, DH, MW, LR, VG-C, and TH acquired the data used in this work; MB and H-WU performed analyses; all authors critically revised the paper for important intellectual content, approved the final version to be published and agreed to be accountable for the accuracy and integrity of any part of the work.

## Conflict of Interest Statement

The authors declare that the research was conducted in the absence of any commercial or financial relationships that could be construed as a potential conflict of interest.
