# Supplementary figures and images for: Classification for Longevity Potential: The Use of Novel Biomarkers
Source: Front Public Health. 2016 Oct 28;4:233. doi: 10.3389/fpubh.2016.00233 (PMC5083840; doi:10.3389/fpubh.2016.00233)

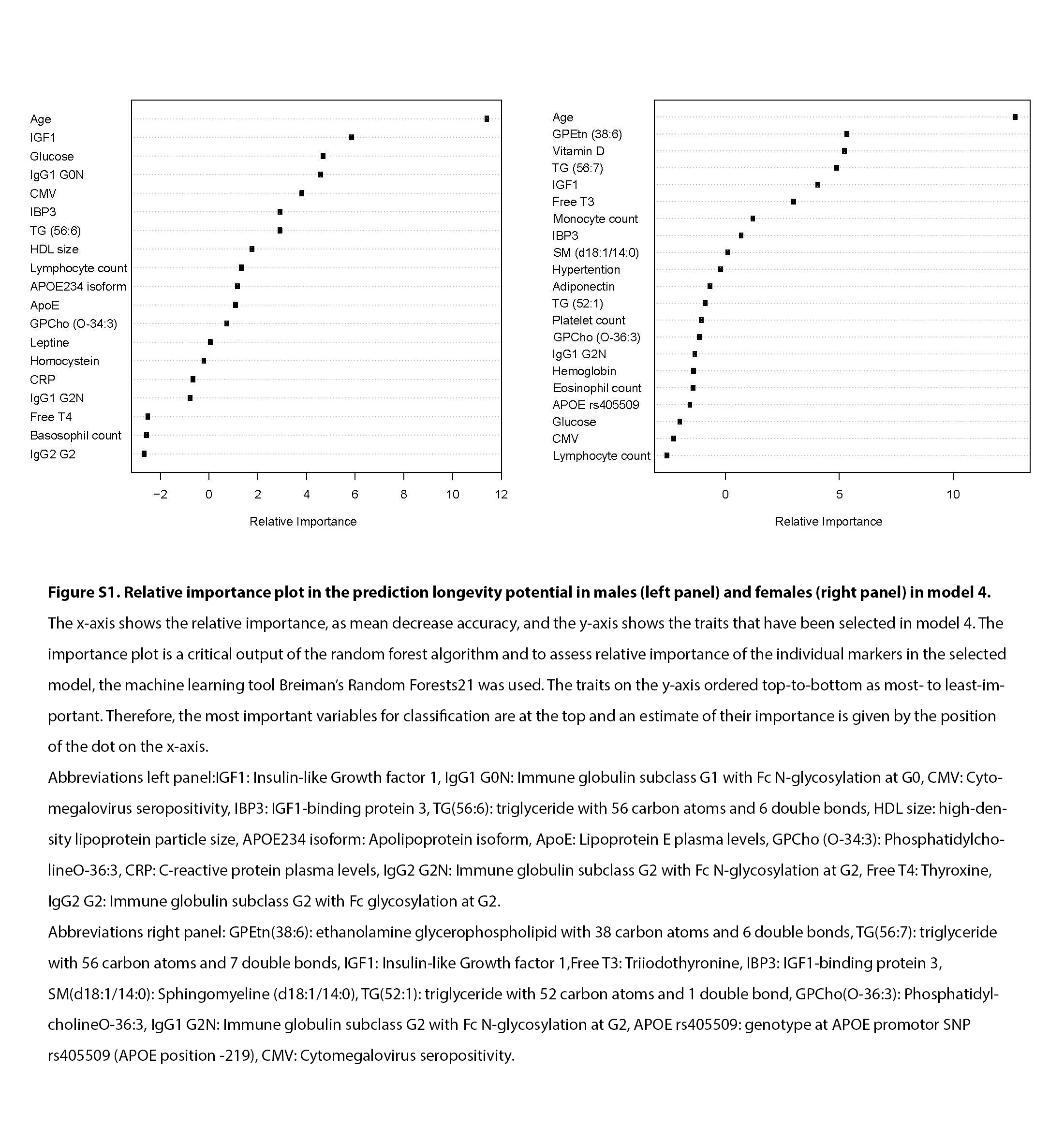

Supplement: Supplementary file 3 [file Image_1.JPEG]
